# Interaction of bacteria and archaea in a microbial fuel cell with ITO anode

**DOI:** 10.1039/c8ra01207e

**Published:** 2018-08-10

**Authors:** Qingqing Jiang, Defeng Xing, Lu Zhang, Rui Sun, Jian Zhang, Yingjuan Zhong, Yujie Feng, Nanqi Ren

**Affiliations:** State Key Lab of Urban Water Resource and Environment (SKLUWRE), School of Municipal and Environmental Engineering, Harbin Institute of Technology, 2nd Campus of HIT Box 2614 No. 73 Huanghe Road Harbin 150090 China rnq@hit.edu.cn; Shenzhen Greenster Environmental Technology Co., Ltd. Shenzhen 518055 China

## Abstract

A microbial fuel cell with an indium tin oxide (ITO) coated glass anode was used to study the mechanism of electricity generation and electron transfer of electrochemically active microbes (EAMs). A simple method of ITO anode pretreatment (pickling) was developed to improve the performance of the microbial fuel cell. After proper treatment, ITO-glass anodes maintained their conductivity with a slight increase in resistance. Using this pickling pretreatment, the ITO-glass microbial fuel cell with an anode area of only 8.3 cm^2^, was successfully initiated and obtained a stable voltage and power output of 418.8 mW m^−2^. The electrode material with pretreatment showed optimal performance for the *in situ* study of EAMs. DNA was extracted from various parts of the reactor and the microbial communities were analyzed. The results indicated that the large proportion of methane-related microbes on the cathode of the MFC was one of the reasons for its high COD removal and low columbic efficiency. ITO glass is suitable as an anode material for the *in situ* study of EAMs, and shows potential for practical application.

## Abstract 2

A microbial fuel cell with an indium tin oxide coated glass anode was used to study the mechanism of electricity generation and electron transfer of electrochemically active microbes.

## Graphical abstract

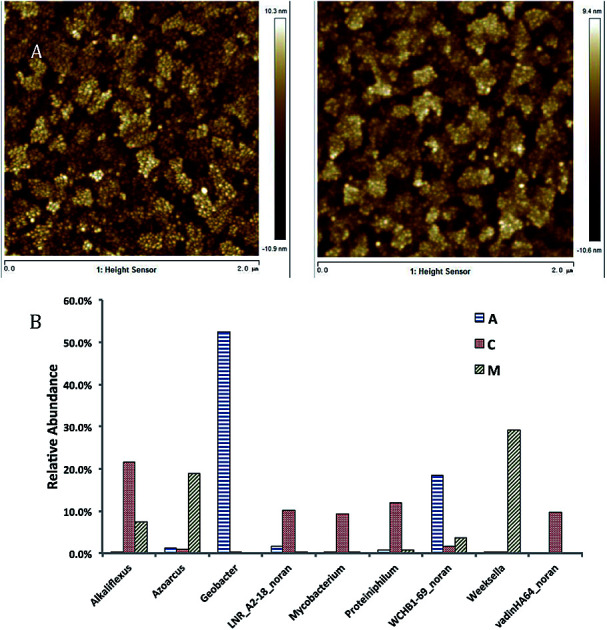

Microbial fuel cells (MFCs) are one type of “green energy generating” device that is able to convert the chemical energy of organic matter into electrical energy.^1^ The process of conversion from chemical energy to electrical energy takes place during wastewater treatment using MFCs. This type of device utilizes microorganisms as the biocatalyst and has attracted a great deal of attention from researchers around the world.^1–4^

Studies of MFCs have been mainly focused in a few specific areas: increasing the efficiency of converting organic matter into electrical energy, reducing the cost of assembling the reactors, and the mechanism of MFCs that converts chemical energy into electrical energy.^2,4–6^

In the first step of energy conversion, microorganisms at the MFC anode consume organic matter from their growth medium and release electrons.^7^ To study this critical step involving the anode, the microorganisms located their, and their mechanism of electron deposition, it is necessary to choose proper anodic materials.^8^

At present, more and more choices have got to fabricate or modify the electrode of MFCs, with nanomaterials, nanocomposites, graphene sheet and graphene oxides owing to their high surface area, porosity and prompt electron conduction properties.^9–13^ The results showed that the electrodes fabricated or modified by nonamaterials exhibited excellent electrochemical activity. Significant redox peaks have found in cyclic voltammograms and electricity production of the MFCs also obtained.

To harvest the largest columbic efficiencies and chemical oxygen demand (COD) removal efficiencies, most MFC anodes are carbon-felt brushes with a large surface area.^1,14^ These types of anodes are three-dimensional and are not easy to observe in their working state or after operation under the microscope.^15,16^ Most electrochemically active bacteria (EAB) are located on the anode, so the observation is critical for the study of the mechanism of electricity generation.^17^ From the level of microbial communities, observing the anode can supplement high-throughput sequencing. From the level of the microstructure of microorganisms, it can provide direct evidence of electrochemically active microbe (EAM) nanowires.^15,16^ To achieve these objectives in this experiment, flat-shaped conductive anodes were used by the researchers.^18,19^

Among the flat anodes with good conductivity, ITO-glass has been widely used in both industrial and scientific applications. ITO-glass is a type of glass that is coated with indium tin oxide, which is one of the most widely used transparent conducting oxides because of its bulk properties, electrical conductivity, optical transparency, and ease in which it can be deposited as a thin film.^15^ With these properties, this material is ideal for observing the growth of EAMs communities. Some studies have already used ITO-glass as the anodes in MFCs for different purposes. To study the bacterial extracellular electron-transfer (ET) respiration of *Shewanella loihica* PV-4, an extensively researched electrochemically active bacterium, the Nakamura and Marsili groups both chose to use a tin-doped indium oxide electrode (ITO) in MFCs as the working electrode.^15,16^ Later, researchers used ITO electrodes to study the mechanism of extracellular electron transfer in the MFCs of pure *Geobacter sulfurreducens* and mixed communities.^16^ To enhance the power output, modifications were made to increase the roughness and conductivity of the ITO electrodes, such as nanostructured polyaniline/titanium dioxide composite, hydroxylated polyaniline, chitosan (CS), α-Fe_2_O_3_ nanoparticles, and others.^20–22^

Most studies have used ITO electrodes with a single culture, while mixed culture grown on ITO electrodes is largely unstudied. In this study, MFCs were operated with ITO electrodes and a mixed culture, laying a foundation for the future study of extracellular electron-transfer (ET) respiration in the multispecies communities.

This low cost and easy to fabricate material was selected for use and a simple pretreatment was conducted to improve its properties. The microbes in the anode, cathode and media were systematically analyzed. The interaction of the different microbes and their effects on electrogenesis are discussed. The results have an important reference both in theoretical and practical fields.

## 1 Materials and methods

### 1.1 Microbial fuel cell construction

In this study, five experimental and one control MFC were constructed. All the MFC reactors used one 330 ml glass bottle as the container. In the experimental MFCs (ITO-MFC), ITO-glass was chosen as the anode. A copper clip, which connected the glass to the circuit, was clipped onto each anode. Each ITO-glass anode was coated on one side with ITO, making only one side of the glass was conductive. This conductive side had an area of 8.3 cm^2^ for each anode. For better adherence, the ITO-glass anodes were pickled by soaking in hydrochloric acid (*V*_HCl_ : *V*_DI water_ = 1 : 2) to increase the roughness of the surface. The duration of anodic ITO-glass pickling treatment with HCl was 0 minute, 1 minute, 1.5 minutes, 2 minutes and 3 minutes, respectively. The control reactor (carbon brush-MFC, CB-MFC) used a carbon felt brush with a diameter of 2.5 cm and height of 6 cm. Cathodes contained 0.5 mg cm^−2^ Pt and three diffusion layers on 30% wt/wt wet-proofed carbon cloth (5 cm^2^, type B-1B, E-TEK).^23^ The anode and the cathode were connected to a 1000 ohm external resistor by a titanium wire in each reactor.

### 1.2 MFC operation

The inoculum used for each reactor was 10 ml sludge from the second sediment tank of the Harbin Wenchang sewage wastewater treatment plant. The feed solution for each reactor was 50 mM PBS with 2 g L^−1^ sodium acetate.^1^ Reactors were operated in batch mode, and 2/3 of the medium in the bottle was replaced the new medium when the voltage fell below 50 mV. All of the experiments were carried out in a 30 °C thermostatic chamber.

### 1.3 Analyses and calculation

The voltage across the external resistance (*R* = 1000 ohm) was measured and recorded by a data acquisition system (2700 Keithley Inc.). Polarization curves were obtained by changing external resistance from 100 ohm to 2000 ohm. Current density (*I*, mA m^−2^) and power density (*P*, mW m^−2^) were normalized to the cathode projected area (*A* = 0.0005 m^2^) and calculated as *I* = *U*/*RA* and *P* = *IU*, respectively. The COD of liquid samples was measured according to standard methods.^24^ The internal resistance of the reactors was measured by EIS using an electrochemical workstation (Autolab Inc. USA), and the data was fitted with the supporting software NOVA (Autolab Inc., USA). All the samples (such as COD, resistance, voltage, roughness, *et al.*) were measured 3 sets of data and took the average.

The coulombic efficiency (CE) is defined as the ratio of the total charge transferred from substrate to anode to the maximum possible coulombs produced if all of the substrate were converted to current.^25^ The total coulombs obtained were determined by integrating the current over time so that the coulombic efficiency (CE) for an MFC run in fed-batch mode, evaluated over a period of time *t*, was calculated as1
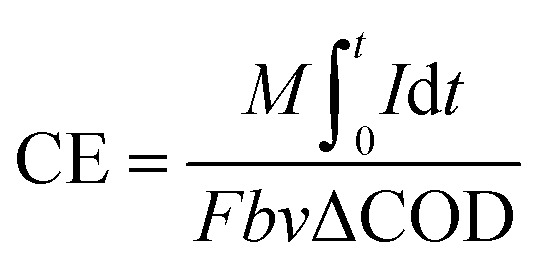


In this study, CE was calculated as2
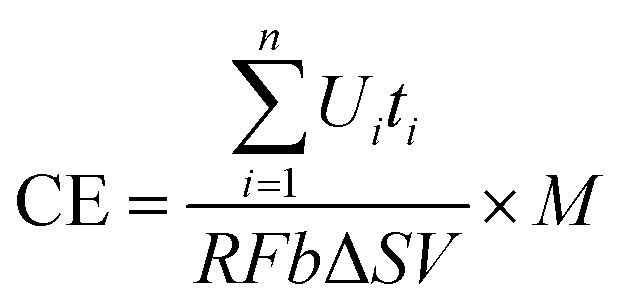
where *U*_*i*_ is the voltage of the MFC over time *t*_*i*_; *M*, the molecular weight of oxygen, is 32; *R* is the resistance; *F* is Faradays constant (96 485 C mol^−1^); *b* is the number of electrons exchanged per mole of oxygen, 4; *V* is the volume of liquid in the anode compartment; and Δ*S* is the change in COD.

### 1.4 Microbial community structure analysis

After 73 days of operation, the ITO-MFC (2 minute pickling anode) with the highest voltage and power density was chosen to be sampled for microbial community analysis. Biofilms on the anode, cathode and the medium of the reactor were collected for high throughput sequencing. Total genomic DNA was extracted by using a DNA isolation kit (PowerSoil DNA Isolation Kit, MoBio Laboratories Inc.) according to the manufacturer's instruction manual. Biofilms on the electrodes were gently rinsed with deionized water (DI water) to remove the residual components. The biofilms were attached tightly to both the anode and cathode, and sterile blades were used to harvest all the biofilms on the surfaces for DNA extraction. Unlike the conventional method of collecting biofilms from several positions of a carbon brush, both the anode and cathode used in the experiment were planar, and the biofilms on them could be fully collected by sterile blades. After confirming the existence of the target DNA by agarose gel electrophoresis and PCR, DNA samples were analyzed by miseq sequencing (Miseq sequencing system, Illumina, America) to analyze the bacterial and archaeal communities.

## 2 Results and discussion

### 2.1 Reactor performance

Except for the MFC with anode pickling time of 3 minutes, all the other MFCs were started successfully. The start-up time of the ITO-MFCs (40 hours on average) was a little longer than that of the control, CB-MFC (30 hours). The ITO-MFC then reached steady electricity output. The highest peak voltage of the ITO-MFCs went as high as 471 mV, slightly lower than that of the CB-MFC (496 mV). After the first refill of the MFCs (200 hours), there was an apparent instability to the ITO-MFC voltage, while the CB-MFC voltage recovered quickly to 490 mV and then ran smoothly. The reason that the MFC with 3 minute anode pickling failed to start was that pickling for too long caused little of the ITO film to remain, so the anode was not suitable for microbial growth. With increasing pickling time, the peak voltages of MFCs with ITO anodes also increased, as shown in Table 1. The MFC with ITO pickling at 2 minutes had the highest voltage among all the ITO-anode MFCs, so the following study focused on the MFC with 2 minute pickled ITO anode.

**Table 1:** The performance of microbial fuel cells

Anode	0-ITO	2 min-ITO	Carbon brush
Anode roughness (nm)	3.28 ± 0.13	4.68 ± 0.21	Not tested
Peak voltage (mV)	335	471	496
Power density (mW m^−2^)	101 ± 13	420 ± 27	655 ± 31
Anode resistance (ohm)	50 ± 1	53 ± 1	<10
Reactor resistance (ohm)	162 ± 8	129 ± 11	106 ± 17
COD removal efficiency	35 ± 3%	56 ± 5%	82 ± 6%

**Fig. 1:**
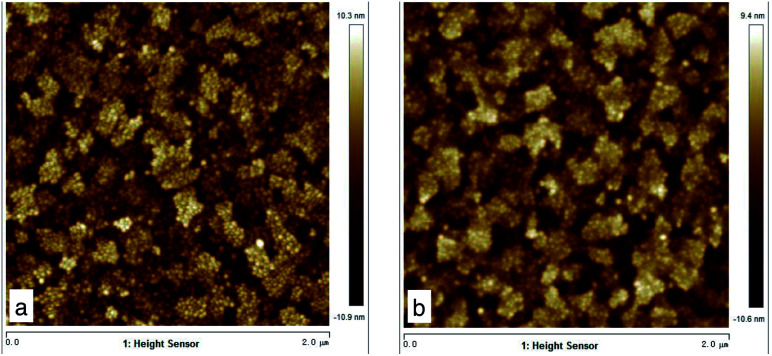
Atomic force microscopy of pickled ITO-glasses. (A) Left: pickled 0 min ITO, roughness = 3.28; (B) Right: pickled 2 min ITO, roughness = 4.68.

The COD removal efficiency of the 2 minutes pickled ITO-MFCs was 56%, much lower than that of the CB-MFC (82%), but higher than that of none-treated ITO anode MFC(35%) (Table 1). The 2 minute pickled ITO anode had a higher open circuit peak voltage (471 mV) and power density (420 mW m^−2^) than those of the ITO-MFC without treatment (peak voltage 350 mV, power density 420 mW m^−2^) (Fig. 1). All the columbic efficiencies were less than 3% in the reactors. Before the operation, the resistance of the 2 minutes pickled ITO-glass (53 ohms) was greater than that of the none-treated anode (50 ohms). After 73 days of operation, the ohmic resistance of the ITO-MFCs (129 ohms) was smaller than the ITO-MFC without anode treatment (162 ohms) (Table 1).

The ITO-MFC did not match the CB-MFC in peak voltage, power density, or COD removal. However, this difference in performance was likely due to the large difference in the surface area of the anode for the ITO-MFC and the CB-MFC. The surface area of the anode was only 8.3 cm^2^ for the ITO-MFC. Considering the carbon felt brush as a cylinder, the specific bristle area was calculated as the external area of each bristle multiplied by the number of bristles per brush and by the total number of brushes.^14^ The external area for the carbon brush was 166.8 cm^2^, 20 times higher than that of the ITO-glass. If the calculation is based on the field of the bristles, the surface was hundreds of times larger than that of the ITO-glass. This result showed that the ITO-glass anode had a better ability to produce electricity than the carbon felt brush with the same anode surface area. The ITO-MFC has a great potential for performance improvements by increasing the surface area of the anode.

**Fig. 2:**
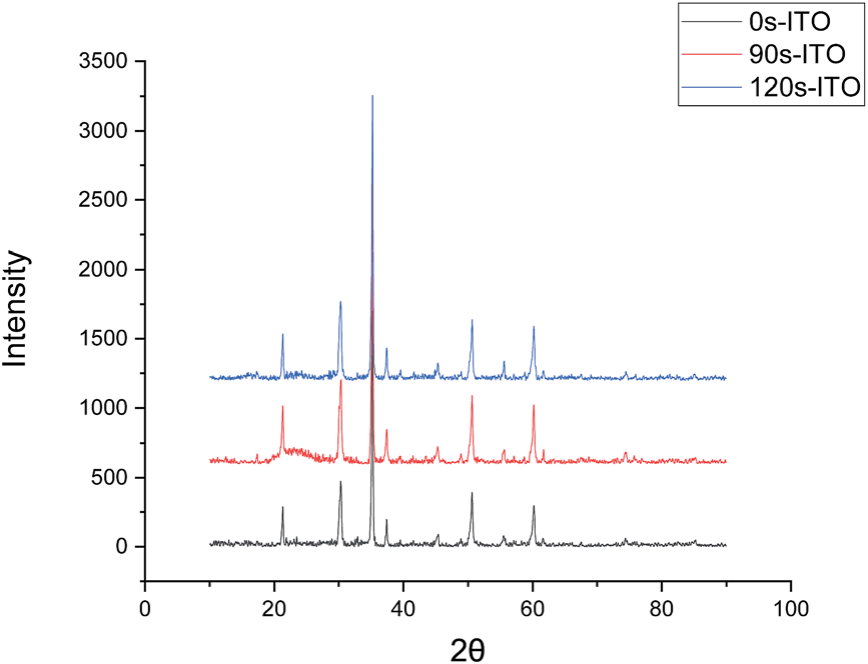
X-ray diffraction patterns of ITO surface with different pickling time. 0s-ITO (Black line): none treated; 90s-ITO (Red line): pickling time = 1.5 min; 120s-ITO (Blue line): pickling time = 2 min.

The surfaces of the ITO-glass before and after pickling treatment were observed by AFM. After the pickling, the roughness of ITO-glass increased from 3.28 to 4.68 (Fig. 1), that means the surface of ITO-glass became easier for microbes to attach. At the same time, pickling did not change crystal structure of the ITO-anode, the results of the X-ray diffraction did not had variations after pickling treatment (Fig. 2).

**Fig. 3:**
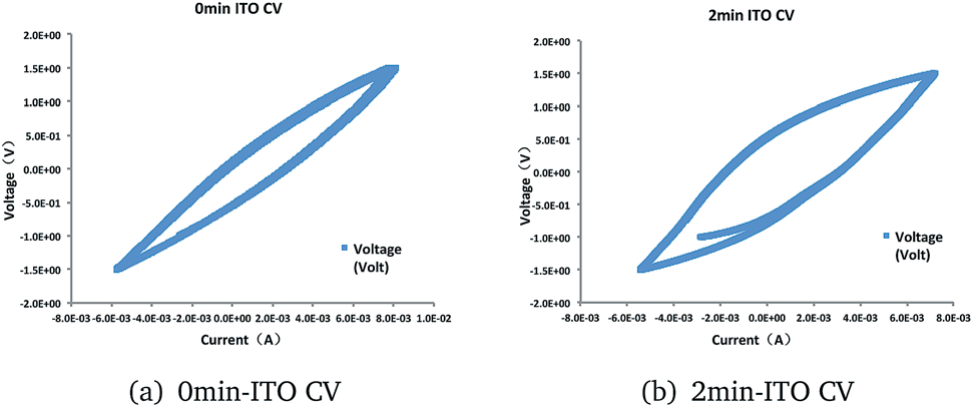
Cyclic voltammograms of (a) 0 min-ITO (b) 2 min-ITO, obtained under working state at a scan rate of 5 mV s^−1^.

No significant redox peaks were observed for the ITO glass. However, the pickling treatment showed higher electricity current. This result indicated that the absence of electrocatalytic activematerial in the ITO glass before treatment. The increase in electricity current represent higher electrocatalytic activity (Fig. 3).

Relative works using flat anode in microbial fuel cells were shown in Table 2. Data shows that MFC with acid pickled ITO anode had larger current than other modification to the flat anodes. Potentiostat-controlling to the anode could also give the EAMs a better environment to grow, so the MFC of *G. sulfurreducens* with +0.24 V potentiostat-control *vs.* SHE. had the highest current among all the works. Among all the works listed, our treatment is easy, efficient and low cost.

**Table 2:** Relative works of flat anode MFCs.(current unit:μA cm^−2^; and the voltage under treatment was potentiostat-controlled *vs.*.SHE)

Anode	Treatment	Bacteria	Substrate	Current
ITO^26^	α-Fe_2_O_3_ nanorod and chitosan (CS)	*Shewanella* PV-4	Sodium acetate	4.4
ITO	Acid pickled	Mixed-cultured	Sodium acetate	120
FTO^27^	TiO_2_	*C. vulgaris*	3N-BBM + V	13
ITO^28^	+0.20 V	*S. oneidensis* MR-1	Lactate	2.6
ITO^16^	+0.20 V	*S. loihica* PV-4	Lactate	3.7 ± 1.5
ITO^15^	+0.24 V	*G. sulfurreducens*	Acetate	150

Based on these findings and relative works shown in Table 2, the MFC with ITO anode is suitable for *in situ* analysis of EAMs. The ITO glass could be taken off the reactor and observed by optical microscope and AFM at any time, as well as undergo DNA extraction and analysis, then replaced to continue the experiment. The formation and growth process of EAM biofilms could be investigated in this way. With the performance of the MFC with ITO anode already improved by the pickling treatment, further improvement and real applications may be possible.

### 2.2 Bacterial community analysis

An alpha diversity analysis (Table 3) of the anode, cathode, and planktonic bacteria grown in the medium for the ITO-MFC indicated 128 (anode), 133 (cathode) and 119 (medium) operational taxonomic units (OTUs) based on a minimum 97% identity criteria. The total number of OTUs and species richness estimated by using the Chao1 estimator was 142 (anode), 136 (cathode) and 136 (medium); the ACE estimator was 141 (anode), 137 (cathode) and 130 (medium) with infinite sampling. These results indicate that among the different positions, the anode samples had the highest richness while the medium samples had the lowest.

**Table 3:** Similarity-based bacterial OTUs, species richness, and diversity estimates were obtained by setting a distance of 0.03

Sample	Reads	OTUs	ACE	Chao 1	Shannon
ITO-MFC-anode	16 013	128	141	142	2.03
			(134, 159)	(133, 168)	(2, 2.06)
ITO-MFC-cathode	16 052	133	137	136	3.13
			(134, 147)	(134, 147)	(3.1, 3.15)
ITO-MFC-medium	11 918	119	130	136	2.68
			(123, 147)	(124, 172)	(2.65, 2.71)

The Shannon diversity index accounts for the relative abundance and evenness (how evenly the abundance of different species is distributed) of the species or OTUs present in the community.^29^ The cathode had the highest diversity (Shannon = 3.13), the anode biofilm had the lowest diversity (Shannon = 2.03), and the medium sample was in between (Shannon = 2.68).

**Fig. 4:**
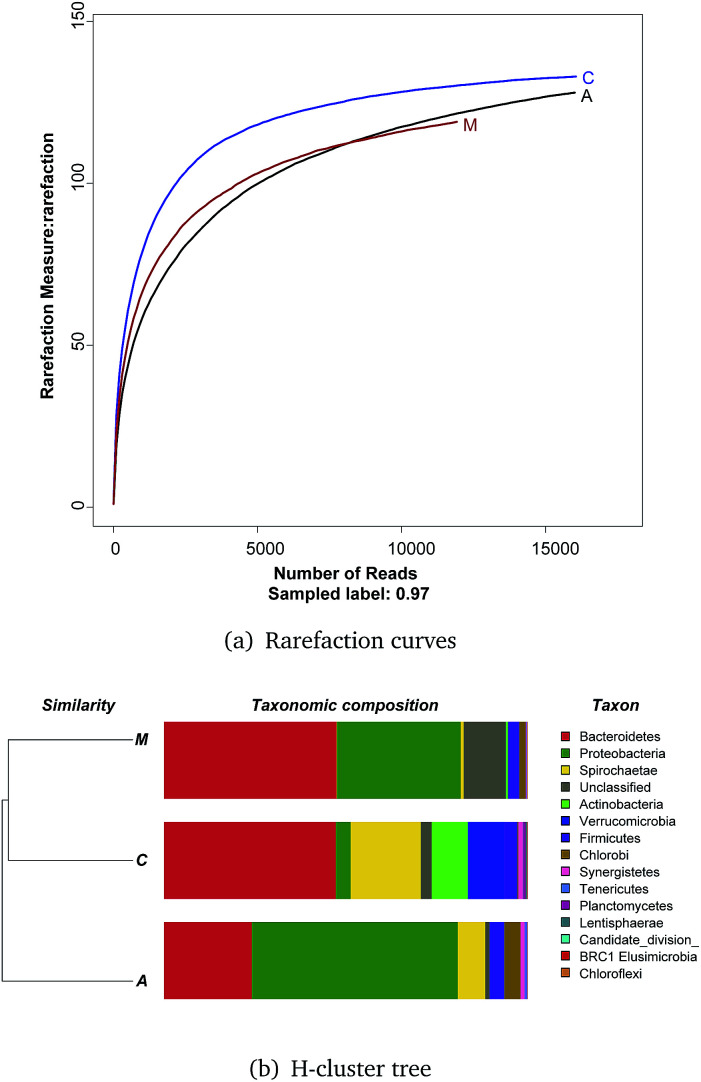
Rarefaction curves and H-cluster tree of bacterial OTUs generated from DNA samples of the ITO-MFC reactor. (A) Rarefaction curves of bacterial OTUs generated from DNA samples of the ITO-MFC reactor based on 97% similarity.(B) H-cluster tree of bacterial communities, A = anode, C = cathode, M = medium.

Rarefaction curves (Fig. 4a) based on a 97% similarity criteria of the 16S rRNA gene sequences reached a plateau, meaning few OTUs continued to emerge for samples after 10 000 reads.

An H-cluster tree used for analyzing huge datasets was employed to identify the differences of the three bacterial community structures based on their phylogenetic lineages (Fig. 4b). This method is effective in revealing ecological patterns between samples.^30^ The cathode and medium communities were closer together based on dendrogram cluster analysis.

**Fig. 5:**
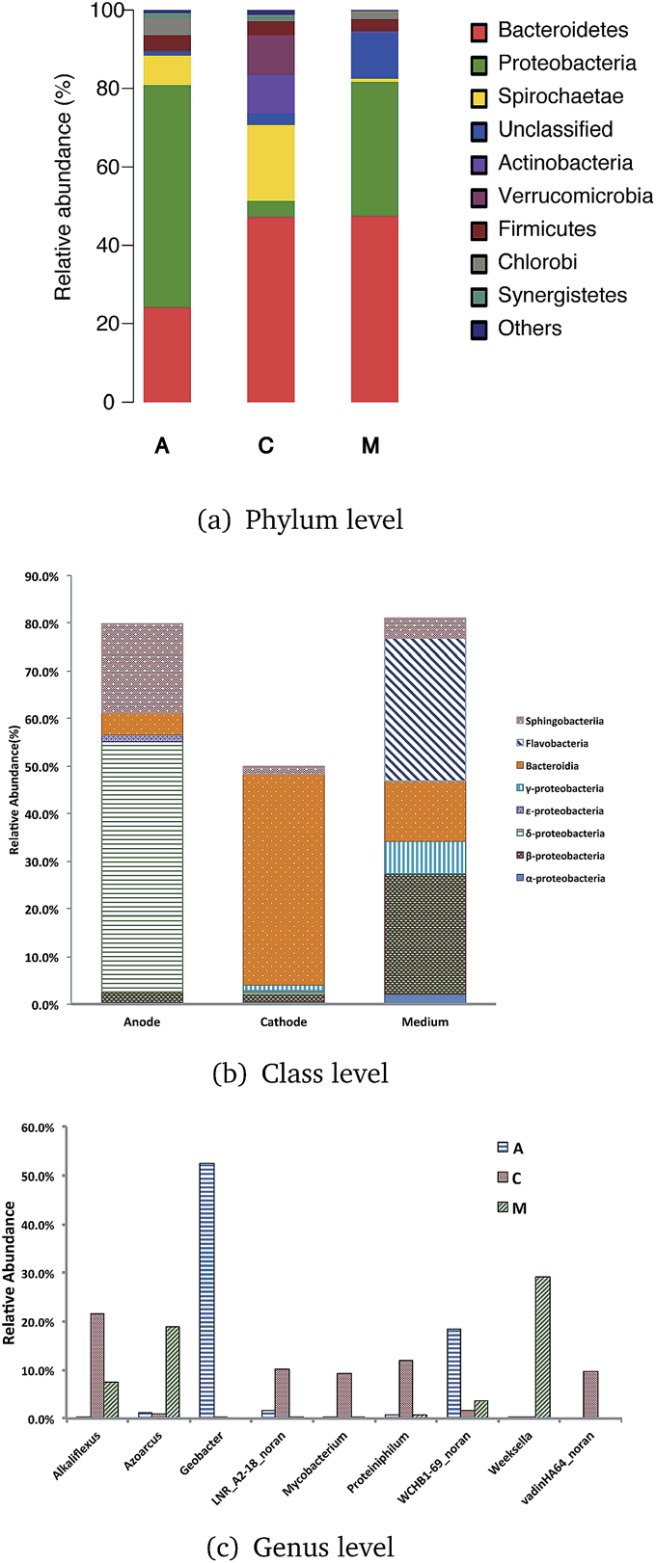
Taxonomic classification of bacterial DNA sequences from communities of ITO-MFC at the (A) phylum (B) class level and (C) genera level, and the distribution of the most dominant phyla of Proteobacteria and Bacteroidetes.

**Fig. 6:**
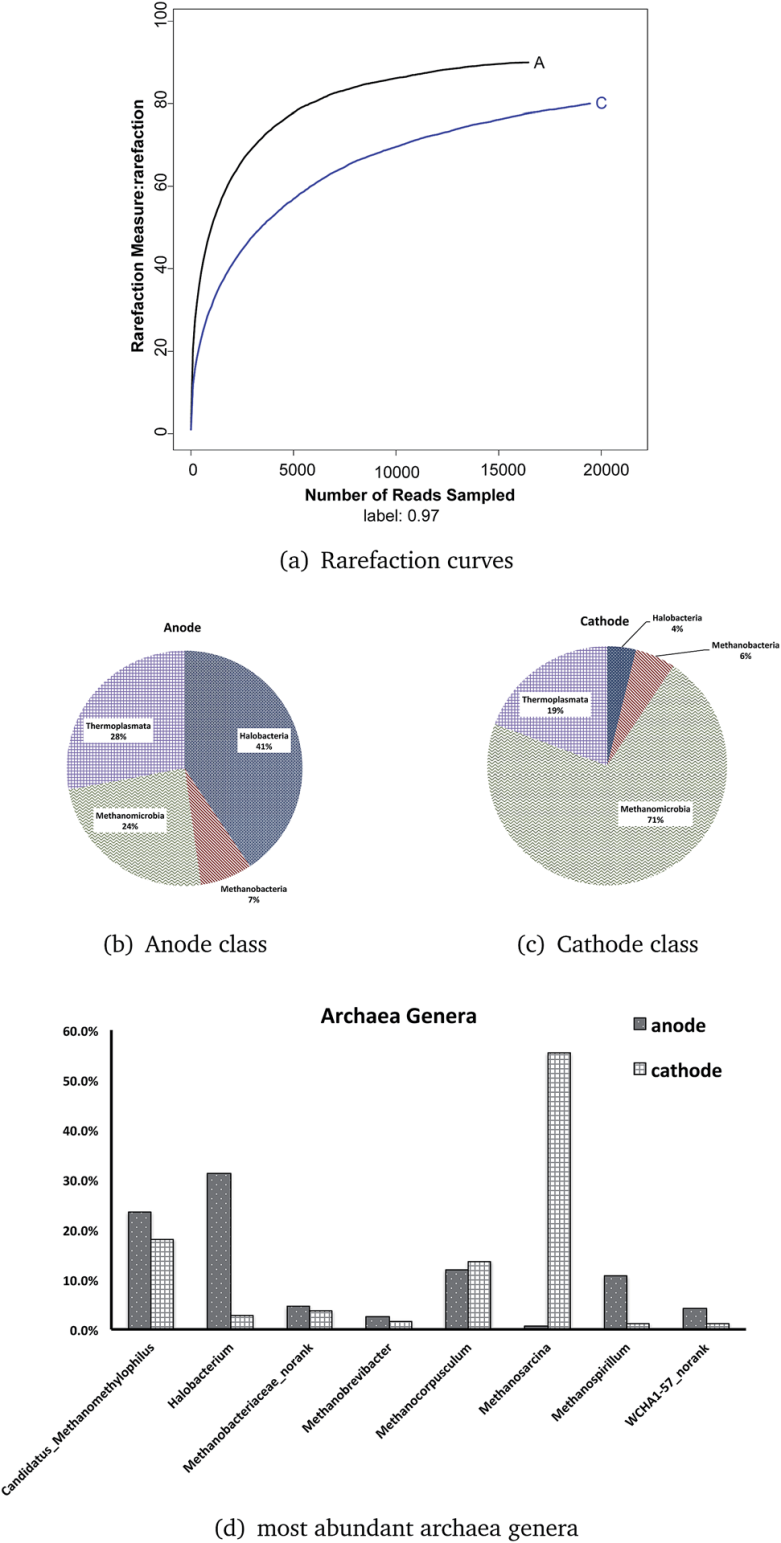
(A) Rarefaction curves of archaeal OTUs generated from DNA samples of the ITO-MFC reactor. A = anode, C = cathode. (B) and (C) Taxonomic classification of archaeal DNA sequences from communities of the ITO-MFC at the class level. (D) The most abundant eight archaea genera of the anode and cathode.

Qualified sequences were assigned to known phyla, class, and genera (Fig. 5) to identify the phylogenetic diversity of the bacterial communities. A total of 14 identified phyla were observed, indicating high biodiversity, which was consistent with Shannon index estimation. Six phyla with greater than 1% abundance are shown in Fig. 6a. Bacteroidetes were the most abundant population among the three communities (anode 24.1%, cathode 47.2%, and medium 47.5%), followed by Proteobacteria (anode 56.7%, cathode 4.1%, and medium 34.1%). Firmicutes were roughly equivalently proportioned among the three positions in the reactor (anode 4%, cathode 3.6%, medium 3.1%). Spirochaetae occupied 19.3% in the cathode, while only 7.6% in the anode and 0.8% in the medium. The microbes that mainly grew on the cathode were Actinobacteria (9.9%) and Verrucomicrobia (10%) and were difficult to find in the other positions.

Over 50% of the OTUs were in the phyla Bacteroidetes and Proteobacteria. Classes were analyzed in these two phyla. The anode biofilm had the largest portion of δ-proteobacteria (52.6%); the microbes on the cathode had the greatest proportion of Bacteroidia (44.3%), and the planktonic bacteria in the medium held a significant amount of *Flavobacteria* (30.0%).

More than 80 genera were revealed in sequencing, and the nine most common were *Geobacter*, *Weeksella*, *Alkaliflexus*, *WCHB1-69 norank*, *Azoarcus*, *Proteiniphilum*, *LNR A2-18 norank*, *vadinHA64 norank*, and *Mycobacterium*. Among these genera, *Geobacter* had the largest portion both on the anode and in the whole ITO-MFC reactor. *Weeksella*, mostly found in the medium, occupied nearly 30% of the relative abundance in the bacteria growing in the liquid. Over 20% of the microbes on the cathode were *Alkaliflexus*, which could also be found in the medium (7.4%).

*Geobacter* is the most common genus generating electrons in MFCs, MECs and MDCs.^2,5,6,31,32^*Verrucomicrobia*, a genus from phylum Verrucomicrobia, which could only be found in the cathode, is a methanotrophic bacteria.^33^ This result indicated that there was some methane produced near the cathode of the ITO-MFC, and might be in balance with the methane producing archaea. There are also some microbes that could utilize methane in the environment belonging to the phylum Proteobacteria.^34^

Interspecies electron transfer between bacteria is very common. Kato found that sulphur-reducing *Bacillus* and *Thiobacillus* denitrification can promote electron transfer between bacteria by conducting electromagnetic nanoparticles as intermediates and complete the process of acetate oxidation and nitrate reduction. Hydrogen, sulfate, formate and some insoluble substances, such as magnetite, can also be used as electronic shuttle mediators to promote electron transfer between strains. This transfer will increase the concentration of electrons in the system, thus improving the efficiency of the MFC. In addition, some microbes can synthesize mediators, such as *Pseudomonas aeruginosa* strain KRP1, which can synthesize chloropyrazin and phenazine-1-formamide, and its synthesis is not only used by itself, but other microbes can also use its mediator to transfer electrons from it to improve the speed of electron transfer and improve the performance of the reactor. Microbes that can participate in extracellular electron transfer are distributed in almost all bacterial phyla,^35^ especially the abundance of Proteobacteria and Firmicutes in the anode.^36,37^ The most widely studied microorganisms are bacteria of the genus *Shewanella*; *Geobacter sulfurreducens* and *Shewanella oneidensis* MR-1. However, in recent years, a large number of other kinds of microbes have been isolated, and more and more information about metabolic processes and/or genomes has been obtained. The more typical examples are *Pseudomonas aeruginosa*, *Rhodopseudomonas marshes* and *Rhodopseudomonas spalustris*, *Clostridium acetobutylicum* of *Clostridium*, and *Thermincola* sp.^38–41^

The main role of phylum Bacteroidetes in the community is to produce acid by fermentation, and to provide metabolic materials for other electroactive bacteria.

The microbial community of cathodic biofilm is much less than that of the anode. The microbes that play key roles in the biocatalytic process are mainly bacteriobacteria (Bacteroidetes) (*e.g. Sphingobacterium*) and *Proteus* (Proteobacteria) (such as *Acinetobacter*).^42^

### 2.3 Archaeal community analysis

For the archaeal communities, only the biofilms grown on the anode and cathode of the ITO-MFC were tested with pyrosequencing. More high-quality reads were from the cathode (19 456) than the anode (16 450), but more OTUs were tested on the anode (anode = 90, cathode = 80) based on a minimum 98% identity criteria. The total number of OTUs or species richness estimated by using the Chao1 estimator was 92 (anode) and 89 (cathode); while the ACE estimator was 91 (anode) and 88 (cathode) with infinite sampling (Table 4). These results indicated that between the anode and cathode, the anode samples had slightly higher richness than the cathode. The anode had a higher diversity (Shannon = 2.72) than that of the cathode (Shannon = 1.65).

**Table 4:** Similarity-based archaeal OTUs, species richness and diversity estimates by setting a distance of 0.03

Sample	Reads	OTUs	ACE	Chao 1	Shannon
2 min-ITO-anode	16 450	90	92	91	2.72
			(90, 100)	(90, 98)	(2.7, 2.74)
2 min-ITO-cathode	19 456	80	89	88	1.65
			(83, 105)	(82, 109)	(1.63, 1.67)

Rarefaction curves (Fig. 6a) based on 97% similarity of the 16S rRNA gene sequences reached a plateau, meaning the Illumina Miseq sequencing obtained enough reads to conduct the analysis.

There was only one phylum, Euryarchaeota, in the ITO-MFC reactor. Both the anode and the cathode consisted of four classes: Halobacteria, Methanobacteria, Methanomicrobia, and Thermoplasmata (shown as Fig. 6b and c). In the anode, Halobacteria had the highest relative abundance (40.6%), followed by Thermoplasmata with an abundance of 27.9%. Two classes related to methane production occupied the minimum percentage (Methanomicrobia = 24.3%, Methanobacteria = 7.1%). The proportion of the archaeal community at the cathode was quite different from that of the anode. Methanomicrobia had a large proportion of 71.3%, and in combination with another archaeal class, Methanobacteria (5.4%), over 76% of the archaeal microbes on the cathode were related to methane production. Thermoplasmata occupied 19.3% and was the most abundant class of the anode. Halobacteria were only a small amount at 3.9% on the cathode of the ITO-MFC.

Twelve archaeal genera were found on both surfaces of the anode and cathode. The most abundant eight genera (*Candidatus Methanomethylophilus*, *Halobacterium*, *Methanobacteriaceae norank*, *Methanobrevibacter*, *Methanocorpusculum*, *Methanosarcina*, *Methanospirillum*, and *WCHA1-57 norank*) are shown in Fig. 6d. From six to eight genera were related to methane. The genera that occupied more than 10% in the anode are *Halobacterium* (31.3%), *Candidatus Methanomethylophilus* (23.5%), *Methanocorpusculum* (11.9%), and *Methanospirillum* (10.7%), while those in the cathode are *Methanosarcina* (55.4%), *Candidatus Methanomethylophilus* (18.1%), and *Methanocorpusculum* (13.7%). Among all the genera, the most abundant on the anode was the genus *Halobacterium* (31.3%), which occupied only 2.8% on the cathode. And the genus with the greatest abundance on the cathode, archaea *Methanosarcina* (55.4%), was almost not detectable on the anode (0.7%).

From the results, most archaea in the ITO-MFC were methane-related microbes (>60% in the anode, >90% in the cathode). *Candidatus Methanomethylophilus* had similar distributions in the anode (23.5%) and cathode (18.1%). This is a type of a methanogen that is also present in human gut, living under anaerobic conditions with methanol as its substrate.^43^ This indicated there might exist some methanol in the medium from the metabolic activities of the microbes. Similar to *Candidatus Methanomethylophilus*, *Methanocorpusculum* (anode 11.9%, cathode 13.7%) and *Methanobacteriaceae norank* (anode 4.6%, cathode 3.7%) did not have a different distribution between the anode and the cathode.

The species *Methanosarcina mazei* from the genus *Methanosarcina* was contained in considerable proportion on the cathode (55.4%). This methanogenic archaeon is able to thrive on various substrates and therefore contains a variety of redox-active proteins involved in both cytoplasmic and membrane-bound electron transport. This organism possesses a complex branched respiratory chain that has the ability to utilize different electron donors.^44^ One probable reason *Methanosarcina* inhabited the cathode was its consumption of part of the electrons transferred from the outer circuit. The fact that *Methanosarcina* could also use acetate as a substrate might explain why some of the MFCs had a high COD removal efficiency while having a low coulombic efficiency.^45^

Additionally, this species may have direct interspecies electron transport (DIET) with Geobacteraceae.^46^ The enrichment of *Methanosarcina mazei* on the cathode in this reactor is much larger than that of the anode, and it is possible to study the possible preference of *Methanosarcina* in different positions of the same reactor in subsequent studies.

With the large proportion of methane-related production of archaea, this ITO-MFC may have had impressive methane production as a microbial electrolysis cell (MEC). R. Sun *et al.* have used alkaline pretreated waste activated sludge to produce methane, which also had a large abundance of *Methanosarcina* (63.3%) and better methane production than the untreated sludge (*Methanosarcina* 6.4%). Additionally, another study using 454 pyrosequencing on the anode communities in MECs using activated sludge (alkaline pretreated and untreated), found that almost no *Methanosarcina* was found on the anodes.^47,48^ These results are in accordance with data in this experiment from Miseq sequencing.

If MFCs or MECs are used as reactors to produce methane, alkaline pretreated activated sludge as the inoculum has been shown effective at enriching the methane-related archaea.^3,47^ When 2-bromoethanesulfonate was used to restrain methane-related archaea in MFCs, the voltages of the reactors did not have large difference, while the power output of MFCs with 2-bromoethanesulfonate was 30% lower than without.^49^ If MFCs were designed specifically to remove the contaminants in the medium and produce electricity, methane production could enhance the electrical output.

In this experiment, the most abundant archaea on the ITO-glass anode was Halobacteria (40.6%), which has not been well studied in the field of MFCs. Most studies have focused on the methanogens in the bioelectrochemical systems. As archaea are widely distributed in sludge and saline environments, Halobacteria has potential for unconventional sewage degradation under extreme environmental conditions.^50^ Halobacteria have been utilized to deal with the organic contaminants, organophosphorus pollutants, and heavy metal problems of high-salinity wastewater. Most studies concentrated upon analyzing bioelectrochemical phenomenon and strain separation. The study of the degradation mechanism of pollutants might be a key step in developing the industrial potential of Halobacteria.^51^ Abrevaya *et al.* used two strains of Halobacteria, *Haloferax volcanii* and *Natrialba magadii*, in microbial fuel cells to produce electricity; both strains produced electrical power in MFCs, and the efficiencies were enhanced after adding outsourcing mediators.^52^ Combined with our experimental results, Halobacteria can survive on a large scale on the surface of electrodes in the saline environments, and they are likely to cooperate with the electrochemically active bacteria to produce electricity. This discovery provides a new way for selecting archaea to deal with high salinity and high COD wastewater. Whether the Halobacteria archaea collaborates or competes with methanogen archaea needs to be considered for subsequent industrial production of methane. In addition, this experiment has successfully enriched Halobacteria on a planar ITO electrode, and provides a feasible method for the subsequent study of electron transportation mechanisms in Halobacteria.

The study of archaeal communities in the MFCs has not been reported often in the past. However, it is meaningful to investigate the archaeal community of MFC reactors because these microbes not only consume organic matters in the medium but also may take part in the process of electron transmission.

The traditional DNA collecting method for carbon brush electrodes is often sampling several points of the brush to ensure the collected samples are a good representation of the biofilm as a whole. In this study, the experimental anode was a piece of flat ITO glass, and the cathode was carbon cloth. The biofilms were grown on the surface of the planar electrodes, and could be collected for whole community sequencing. DNA samples obtained by this method covered almost all microbes of the biofilm on the electrode surface, and the overall biofilm composition of the electrode was more accurate than that from multi-point collected samples of the carbon brush electrode. In addition, the biofilm grown on the planer ITO glass electrode surface was thick and visible at the time of acquisition. In addition to collecting all the electrode biofilm, the anode can also be divided into several parts according to the distance from the cathode or the circuit, such that different distances can be collected separately. This experiment not only estimated microbe population density by comparing the concentration of DNA after extraction, but also analyzed the composition of biofilms in different locations of the anode. This allowed for the study of the microbial composition of the electrode surface. With *in situ* staining and microscopic imaging techniques, the anode biofilm extraction and composition analysis at specific locations under laser scanning confocal microscopy and atomic force microscopy can also be developed in the future. The microbial fuel cell is a mix-cultured reactor; each part has the distribution of bacterium and archaea. The *Halobacterium* belongs to anaerobic archaea, so its proportion on the anode is much higher than on the cathode. The purple membrane on the cell membrane contains a special photosensitive protein Bacteriorhodopsin (Bacteriorhodopsin, BR), and the light energy can be absorbed in the low oxygen and bright environment. The proton pump is pumping the protons out of the cell. When the proton moves to the cathode, it will produce hydrogen, methane or water depending on the different reducing substrates. Therefore, *Halobacterium* can participate in the metabolism of the electrochemical microorganism. The protons produced by *Halobacterium* may neutralize the electrons produced by the other electroactive bacterium of the anode, consume the substrate of the reactor and do not increase the current intensity in the system, thus reducing the coulombic efficiency of the reactor.

The other archaeal dominant genera were all related to methane production. In other studies, agglomerates of *Methanosarcina mezi* with the electrochemical active bacteria *Geobacter* can be produced. In this symbiosis, the *Methanosarcina* can directly absorb the electrons released by *Geobacter* through conductive pili, and the community does not donate electrons as a whole. However, in this study, *Geobacter* and *Methanosarcina* were in two different parts of the reactor, respectively, and *Geobacter* was mainly located in the anode, which was more than 50% of the bacteria, while *Methanosarcina* was mainly distributed in the cathode, and 55% of the archaea. In addition, it is reported that *Methanosarcina* has the ability to absorb electrons directly from other sources. *Methanosarcina* receives the electron on the cathode, causing the imbalance of the electrons and protons in the system, and cannot restore the oxygen into water, which affects the power production efficiency and consumes a large number of substrates to reduce the coulombic efficiency of the reactor.

It has been confirmed that the methanogenic archaea *Methanosarcina* and *Methanosaeta* are able to carry out direct extracellular electron transfer, and the study of other strains is still in progress. The main microbes that have been found to cooperate with the methanogenic archaea – producing bacteria through direct extracellular electron transfer (DIET) include *Geobacter*, *Pseudomonas*, *Syntrop Homondaceae*, *Syntrophomonas*, *Clostridium*, *Bacillaceae* and so on.

The interaction between methanogens and bacterium is not only electron transportation, but also competition for substrates. Methanogenic archaea of hydrogen nourishment use H_2_, formate and other electronic donors to reduce CO_2_ to produce CH_4_; methanate methanogenic archaea can produce CH_4_ using methyl compounds (such as methylamine, two methylamine, trimethylamine) and methyl sulfide (such as methanethanol and two methyl sulphur), and a nutritive form of acetic acid. Alkanes only use acetic acid to produce CH_4_ and CO_2_. At present, only *Methanosarcina* and *Methanothrix* (instead of previous *Methanosaeta*) can produce CH_4_ by acetic acid.

Our results indicated that the interaction between the archaea and the bacteria is complex and diverse, and the results are of great significance to the efficiency of the reactor's electricity production, inviting further study of the mechanism. Both the anode and cathode played an important role in the ITO-MFC, with both bacteria and archaea participating in the metabolic activities in reactors. The large proportion of methane-related microbes in this system was one of the reasons for its high COD removal and low columbic efficiency.

As a low cost and easy to fabricated material, ITO glass can provide a flat and liable attached surface for anode microbes. ITO anodes could be removed from the reactor in the working state for microscope and AFM observation, then replaced. The anode film could be collected wholly or selected at precise positions to get accurate microbial community information. Thus the MFC with ITO anode offers a method for electrogenesis mechanism research. Its performance is already improved by pickling pretreatment and could be further improved with potential for practical application.

## 3 Conclusion

The MFC with an ITO-glass anode after a new treatment was generated steady electrical current. Despite the smaller surface of the ITO anode, the peak voltage of the ITO-MFC was similar to the CB-MFC used as the control. This ITO-MFC configuration is a good choice for EAM observation and for further study of electricity-production mechanisms in MFCs because of its feasibility, low-cost, and simplicity of anode pretreatment.

DNA analysis of bacteria and archaea showed that both the anode and cathode played an important role in the ITO-MFC, with both bacteria and archaea participating in the metabolic activities in reactors. The microbial communities at different parts of the reactor indicated that the electricity production was related to methane generation. The large proportion of methane-related microbes on the cathode of the MFC was one of the reasons for its high COD removal and low columbic efficiency. ITO glass is suitable as an anode material for the *in situ* study of EAMs, and shows potential for practical application.

## Conflicts of interest

There are no conflicts to declare.

## Supplementary Material
